# Acute Pancreatitis in Inflammatory Bowel Disease: Results from the European Pandora Study

**DOI:** 10.3390/medicina61091532

**Published:** 2025-08-26

**Authors:** Maria Cristina Conti Bellocchi, Martina Cattani Mottes, Andreas Blesl, Anneline Cremer, Stefano Festa, Mathieu Uzzan, Tiago Cúrdia Gonçalves, Antonio Rispo, Chiara Viganò, Ioannis Koutroubakis, Antonietta Gerarda Gravina, Daniela Pugliese, Piotr Eder, Sophie Vieujean, Massimo Claudio Fantini, Clara Yzet, Konstantinos Argyriou, Lieven Pouillon, Davide Giuseppe Ribaldone, Andrea Michielan, Marie Truyens, Anna Viola, Edoardo Vincenzo Savarino, Pierre Ellul, Javier P. Gisbert, Vrakas Spyridon, Michele Campigotto, Raquel Oliveira, Angela Variola, Laura Loy, Aldis Pukitis, Maria Fragaki, Alessia Dalila Guarino, Aikaterini Mantaka, Laura Ramos, Stefano Francesco Crinò, Rachele Ciccocioppo, Luca Frulloni, Pandora Study Group

**Affiliations:** 1 Department of Medicine, Diagnostic and Interventional Endoscopy of the Pancreas, The Pancreas Institute, University Hospital of Verona, 37134 Verona, Italystefanofrancesco.crino@aovr.veneto.it (S.F.C.); 2 Division of Gastroenterology and Hepatology, Department of Internal Medicine, Medical University of Graz, 8036 Graz, Austria; andreas.blesl@medunigraz.at; 3 Department of Gastroenterology, Erasme Hospital, Université Libre de Bruxelles, 1070 Brussels, Belgium; anneline_cremer@hotmail.com; 4 IBD Unit, Department of Gastroenterology, “San Filippo Neri” Hospital, 00135 Rome, Italy; festa.stefano@gmail.com; 5 IBD Unit, Department of Gastroenterology, Mondor Hospital, APHP, 92110 Clichy, France; mathieu.uzzan@aphp.fr; 6 Gastroenterology Department, Unidade Local de Saude do Alto Ave, 4835-044 Guimarães, Portugal; tiagomcg@hotmail.com; 7 Life and Health Sciences Research Institute (ICVS), School of Medicine, University of Minho, 4800-058 Braga, Portugal; 8 ICVS/3B’s PT Government Associate Laboratory, 4710-057 Braga, Portugal; 9 Gastroenterology Unit, Department of Clinical Medicine and Surgery, University Federico II of Naples, 80138 Naples, Italy; antonio.rispo2@unina.it (A.R.); ale.tizi@hotmail.it (A.D.G.); 10 Department of Gastroenterology, and Center for Autoimmune Liver Diseases, European Reference Network on Hepatological Diseases (ERN RARE-LIVER), Fondazione IRCCS San Gerardo dei Tintori, University of Milano-Bicocca School of Medicine, 20126 Monza, Italy; chiara.vigano@hotmail.itù; 11 Department of Gastroenterology, University Hospital Heraklion, 71500 Heraklion, Greece; ikoutroubakis@gmail.com; 12 Department of Precision Medicine, University of Campania “Luigi Vanvitelli”, 80138 Naples, Italy; antoniettagerarda.gravina@unicampania.it; 13 CEMAD—IBD UNIT—Unità Operativa Complessa di Medicina Interna e Gastroenterologia, Dipartimento di Scienze Mediche e Chirurgiche, Fondazione Policlinico Universitario “A. Gemelli” IRCCS, 00168 Rome, Italy; daniela.pugliese@policlinicogemelli.it; 14 Department of Gastroenterology, Dietetics and Internal Medicine, Poznań University of Medical Sciences, University Clinical Hospital, 60 512 Poznań, Poland; piotreder@ump.edu.pl; 15 Hepato-Gastroenterology and Digestive Oncology, University Hospital CHU of Liège, 4000 Liège, Belgium; s.vieujean@chuliege.be; 16 Department of Medical Sciences and Public Health, University of Cagliari, and Azienda Ospedaliero-Universitaria di Cagliari, 09042 Cagliari, Italy; massimoc.fantini@unica.it; 17 Amiens University Hospital, Université de Picardie, 80054 Amiens, France; yzet.clara@chu-amiens.fr; 18 Department of Gastroenterology, University Hospital of Larisa, 41334 Larisa, Greece; kosnar2@yahoo.gr; 19 Imelda GI Clinical Research Center, Imelda General Hospital, 2820 Bonheiden, Belgium; lieven.pouillon@imelda.be; 20 Department of Medical Sciences, University of Turin, 10126 Turin, Italy; davrib_1998@yahoo.com; 21 Azienda Provinciale per i Servizi Sanitari (APSS), Gastroenterology and Digestive Endoscopy Unit, Santa Chiara Hospital, 38122 Trento, Italy; andrea.michielan@apss.tn.it; 22 Department of Gastroenterology and Hepatology, Ghent University Hospital, 9000 Ghent, Belgium; marie.truyens@uzgent.be; 23 IBD-Unit, Department of Clinical and Experimental Medicine, University of Messina, 98125 Messina, Italy; 24 Gastroenterology Unit, Department of Surgical, Oncological and Gastroenterological Sciences, University of Padua, 35128 Padua, Italy; edoardosavarino@gmail.com; 25 Division of Gastroenterology, Mater Dei Hospital, 2090 Msida, Malta; ellul.pierre@gmail.com; 26 Gastroenterology Department, Hospital Universitario de La Princesa, Instituto de Investigación Sanitaria Princesa (IIS-Princesa), Universidad Autónoma de Madrid (UAM), Centro de Investigación Biomédica en Red de Enfermedades Hepáticas y Digestivas (CIBEREHD), 2548 Madrid, Spain; javier.p.gisbert@gmail.com; 27 Department of Gastroenterology, Tzaneio General Hospital of Piraeus, 18532 Piraeus, Greece; sbrakas@yahoo.gr; 28 Dipartimento Universitario Clinico di Scienze Mediche Chirurgiche e della Salute, Università degli Studi di 34127 Trieste, Italy; michele.campigotto@asugi.sanita.fvg.it; 29 Centro Hospitalar Universitário Do Algarve, Unidade De Portimão, 8500-000 Faro, Portugal; fdoliveira.raquel@gmail.com; 30 IBD Unit, IRCCS Sacro Cuore Don Calabria, 37024 Negrar di Valpolicella, Italy; angela.variola@sacrocuore.it; 31 Department of Biomedical Sciences, Humanitas Clinical and Research Center, Istituto di Ricovero e Cura a Carattere Scientifico (IRCCS) Humanitas Research Hospital, 20089 Rozzano, Italy; lalloy712@gmail.com; 32 Center of Gastroenterology, Hepatology and Nutrition, Pauls Stradins Clinical University Hospital, LV-1002 Riga, Latvia; pukitis@latnet.lv; 33 Department of Gastroenterology, Venizeleio General Hospital, 71409 Heraklion, Greece; mgfragaki@yahoo.gr; 34 Department of Gastroenterology, Chania General Hospital, 73300 Chania, Greece; katmant@gmail.com; 35 Gastroenterology Department of Hospital Universitario de Canarias, 38001 Santa Cruz de Tenerife, Spain; laura7ramos@gmail.com; 36 Gastroenterology Unit, Department of Medicine, The Pancreas Institute, University Hospital of Verona, 37134 Verona, Italy; rachele.ciccocioppo@univr.it (R.C.); luca.frulloni@univr.it (L.F.)

**Keywords:** pancreatitis, IBD, Crohn’s disease, ulcerative colitis, autoimmune pancreatitis

## Abstract

*Background and aims*: An increased risk of acute pancreatitis (AP) has been reported in patients with inflammatory bowel disease (IBD), but data on its prevalence, etiology, and outcomes are limited. *Materials and Methods:* A two-step retrospective analysis spanning 10 years (2011–2020) was conducted across 34 European centers. The first step surveyed the prevalence of AP in patients with IBD, while the second gathered data on disease characteristics, etiology, and outcomes. *Results:* The survey found an expected AP prevalence of 1.13% (780/68,989), though only 0.58% (n = 398) met the inclusion criteria. The mean age was 33.6 ± 14.3; 52% were female, and 56.5% had Crohn’s disease (CD). AP was clinically mild in most cases (86.9%). Among 347 patients with available imaging, no alterations were observed in 81 (23.3%), whereas edematous AP was observed in 218 (62.8%). Drugs (mainly azathioprine) were the leading cause (55.3%), followed by biliary (14.8%) and autoimmune (7.8%) causes. In 13.5% of patients, AP was considered idiopathic. During a median follow-up of 67 months [IQR 34–96] from the index episode, recurrence was observed in 13% of patients, and 1.5% developed chronic pancreatitis. CD patients exhibited distinct risk profiles, including ileal involvement and smoking, whereas ulcerative colitis (UC) patients showed more frequent autoimmune and idiopathic etiologies. *Conclusions:* The PANDORA study established a 0.58% prevalence of AP in IBD patients, which was lower than expected. AP is usually mild both clinically and radiologically. An ileal location in CD and extensive colitis in UC are usually reported, and azathioprine seems to be the most common cause of AP in this setting, especially a few weeks after its introduction.

## 1. Introduction

A wide range of pancreatic disorders has been documented in patients with inflammatory bowel disease (IBD), including Crohn’s disease (CD) and ulcerative colitis (UC). These disorders range from clinically silent elevation of pancreatic enzyme levels to acute pancreatitis (AP), which may or may not progress to chronic pancreatic disease [1]. AP is the most prevalent pancreatic manifestation in IBD patients, with the risk being twice as high in patients with UC and four times higher in patients with CD compared to the general population. The primary causes of AP are gallstones and medications, while alcohol-related AP appears to be less frequent in patients with IBD [2]. The role of IBD medications in inducing AP has been established for azathioprine (AZA), with its effects attributed to hypersensitivity reactions or direct toxicity [3,4]. At the same time, the association with mesalamine, steroids, metronidazole, and sulfasalazine remains a subject of debate [4]. In cases of suspected drug-induced pancreatitis, diagnostic criteria include not only the temporal relationship between drug administration and the onset of AP but also symptom resolution following drug discontinuation and recurrence of AP upon drug re-exposure [5]. Autoimmune pancreatitis (AIP) is an inflammatory disease of the pancreas that is highly responsive to steroids and is characterized by specific radiological features and an optimal response to steroid therapy. Two types of AIP, type 1 and type 2, are known, with different clinical courses, biochemical markers, histological patterns of inflammation, and clinical outcomes [6]. In type 2 AIP, an association with inflammatory bowel disease has been found in 15–45% of patients [7]. Additionally, metabolic factors and conditions such as duodenal obstruction or papillary inflammation are less frequently observed [8]. Currently, it is difficult to compare existing studies due to the absence of standardized pancreatic diagnostic protocols, management strategies, and follow-up procedures, as well as the independent progression of intestinal and pancreatic disorders [9].

## 2. Materials and Methods

### 2.1. Study Design

The PANDORA (PANcreatic DisOrders and Acute Pancreatitis Registry) study consisted of two phases. During the first phase, a snapshot study (named “survey” below) on the prevalence of pancreatic disorders in patients with IBD was conducted. The survey (Appendix A) was sent to participating centers to provide data on the local standard of care and case volumes. In the second phase, centers were granted access to REDCap, a secure web application for building, designing, and managing online surveys and databases. Using REDCap, a detailed electronic case report form (eCRF) was employed to “pseudo-anonymously” collect information concerning patients with pancreatic disorders who were referred between January 2011 and December 2020. This 10-year study period was selected to ensure robust prevalence estimates and establish a temporal cut-off, facilitating a minimum 2-year follow-up. The local Ethics Committee at each participating center approved the study protocol (Prog. N.3767CESC, 20 April 2022). The study was also disseminated via the European Crohn’s and Colitis Organisation (ECCO) platform following review by its Clinical Research Committee.

### 2.2. Patients

Patients with IBD who experienced one or more AP episodes and were admitted to one of the participating centers were retrospectively identified. Each center provided data on the total number of IBD patients managed during the study period and details on the methodology of data collection—whether through a prospectively maintained database or a retrospective review of both digital and paper-based medical records—via the questionnaire (see Supplementary Materials). The study flowchart is presented in Supplementary Figure S1.

The inclusion criteria required were a confirmed diagnosis of UC or CD based on widely accepted criteria 10: age ≥ 18 years and an AP diagnosis according to the Atlanta criteria [10]; (i) severe and persistent abdominal pain; (ii) serum lipase or amylase levels of at least three times the upper normal limit; and (iii) characteristic imaging findings on computed tomography (CT), magnetic resonance imaging (MRI), or ultrasound. Moreover, the severity of AP was scored as mild (no organ failure or complications), moderate (transient organ failure and complications without lasting organ failure), and severe (persistent organ failure affecting one or more organs) [10]. In the case of AIP, the definitive or probable diagnosis was made considering the International Consensus Diagnostic Criteria (ICDC), and the cases were classified as type 1, type 2, or no other specified (NOS), and based on focal or diffuse pancreatic involvement [7]. Local study coordinators ensured that signed informed consent for the use of personal data was obtained from each participant when required by local EC rules.

### 2.3. Study Aims

The primary aim of this study was to estimate the prevalence of AP (calculated as the number of AP cases divided by the total number of IBD patients observed during the study period) in our setting, as reported in the survey. The secondary aims included exploring the etiology of AP, assessing the relationship between IBD medications and AP, evaluating the role of various imaging modalities in enhancing AP diagnosis, and analyzing clinical outcomes, including recurrence rate, evolution to chronic pancreatitis, pancreatic cancer, and death.

### 2.4. Sample Size and Statistical Analysis

Since the study aimed to collect data for a European AP registry including IBD patients, no a priori sample size was estimated. However, based on a Spanish multicenter study 11, which recruited 12,100 IBD patients and found a 1.52% prevalence of AP, the current study anticipated that data would be collected from approximately 25,000 IBD patients. This would provide a 95% confidence interval ranging from 1.4% to 1.7%. The results were summarized using descriptive statistics [mean ± standard deviation (SD) or median with interquartile range (IQR) for continuous variables and frequency distributions for categorical variables]. When appropriate, the χ^2^, Fisher’s exact tests, t-test, or Wilcoxon rank-sum (Mann–Whitney) test were used to compare categorical and continuous variables between independent groups. The McNemar test was used to compare categorical variables within groups, while the paired t-test or the Wilcoxon signed-rank test were applied to continuous variables within groups. All analyses were two-tailed, with a value of *p* < 0.05 considered statistically significant.

## 3. Results

**Figure 1:**
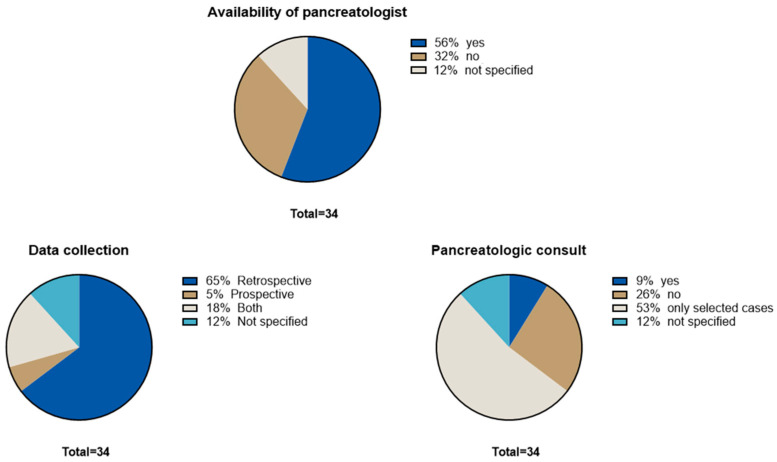
**Pie charts representing the survey results.**

Overall, 34 IBD centers participated in the study. Based on the survey, among 68,989 IBD patients managed during the study period, the prevalence of AP was 1.13% (n = 780). During the second phase of the study, 701 were entered into the database, resulting in an AP prevalence of 0.73%. After a central review of all the eCRFs by two expert pancreatologists (MCCB and LF) and strictly applying the inclusion criteria of the Atlanta classification [10], the prevalence of AP patients dropped to 0.58% (n = 398), despite the availability of a pancreatology consultant in most centers. The results of the survey are presented graphically in Figure 1.

Among the 398 patients with AP, 225 (56.5%) were affected by CD and 173 (43.5%) by UC. Fifty-two percent were females, and the average age at IBD diagnosis was 33.6 ± 14.3 years, with no significant difference between CD and UC patients (*p* = 0.169). The clinical features according to the Montreal classification are shown in Table 1.

In contrast to alcohol consumption, which was not significantly reported, smoking habits and a family history of IBD or pancreatic disease were more commonly observed in the CD group (*p* < 0.00001). Ileal involvement was present in 90.3% of CD patients (with colonic involvement in 49.3%), and the most common disease behavior was non-stricturing and non-penetrating (56%). Among UC patients, extensive disease was the most frequent presentation (59%). Perianal disease was noted in only 11% of cases.

An extraintestinal manifestation (EIM) was reported in 113 patients (28.4%), with 73 in the CD group and 40 in the UC group (*p* = 0.04), with arthropathy being the most common (67 patients, 16.9%) (Supplementary Figure S2).

IBD-related surgery was reported in 100 out of 398 patients (25.1%), of whom 78 had CD and 22 had UC (*p* < 0.00001). The onset of AP occurred immediately after surgery in seven cases (7%). In 50 patients (50%), surgery had been previously performed, with a median time of 62.5 months [IQR 23–121.5], and in 60%, it was an ileocecal resection. The mean age at the onset of AP was 37.6 ± 15.3 years. In most cases, no identifiable risk factors for AP were present, with 69.8% of patients being non-smokers or ex-smokers and 66.6% reporting no alcohol consumption. However, smoking at the onset of AP was more commonly observed in patients with CD compared to UC. In UC patients, AP was more likely to precede the diagnosis of IBD, with a median interval of 3 years [IQR 2–4]. The baseline characteristics of patients at the onset of AP are detailed in Table 2.

**Figure 2:**
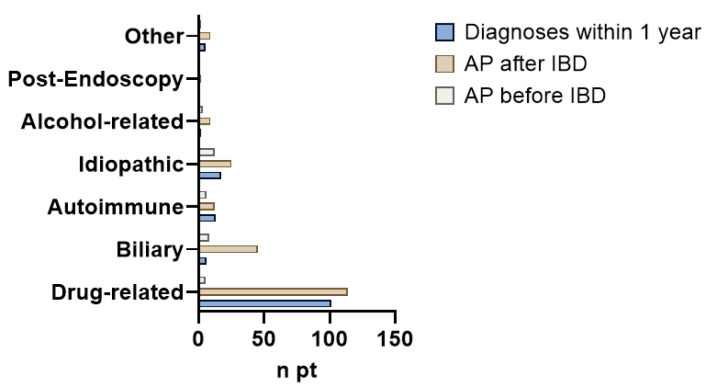
**Column chart representing the etiology of IBD in three groups of patients with different intervals between acute pancreatitis (AP) and inflammatory bowel disease (IBD) diagnosis.**

In 145 patients (36.4%), AP occurred within one year of the diagnosis of IBD, and it was mostly drug-induced (n = 101, 69.6%). In 37 patients (9.3%), the AP episode preceded IBD diagnosis with a median time of 3 years [IQR 2–4], and it was classified as idiopathic in 12 cases (26.6%). This pancreatic and intestinal disease sequence was significantly more frequent in UC patients (*p* = 0.001). In 215 patients (54%), AP onset followed IBD diagnosis, with a median time of 6 years [IQR 2–13]. Figure 2 reports the description of AP etiology related to the diagnosis intervals.

**Figure 3:**
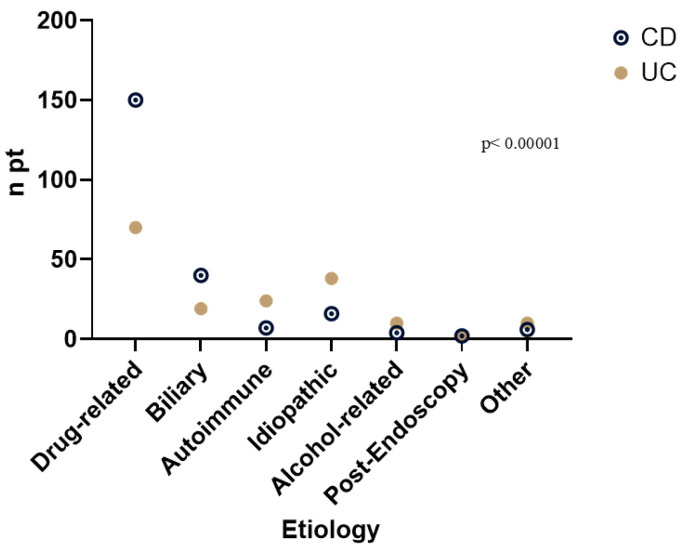
**Different etiology of acute pancreatitis in Crohn’s disease and ulcerative colitis.**

The most common cause of AP was drug-related (n = 220, 55.3%), followed by biliary causes (n = 59, 14.8%). In 54 patients (13.6%), the cause remained unidentified, while AIP was diagnosed in 31 patients (7.8%). Alcohol-related AP was identified in 13 cases (3.5%). A significant difference was observed between CD and UC patients (*p* < 0.00001), with drug-induced AP being more prevalent in CD patients. In contrast, idiopathic AP and AIP were more frequently seen in UC patients. The different percentages are presented in Figure 3.

In patients with a previously or simultaneously diagnosed IBD at the onset of AP, active IBD was reported in 179 out of 361 patients (49.6%); ongoing therapies included 5-ASA (5-aminosalicylic acid) in 153 (42.4%), AZA/6-MP (6-mercaptopurine) in 172 (47.6%), steroids in 48 (13%), and anti-TNF in 57 (15.5%) cases. In 20 (5%) patients, other drugs were reported, including ustekinumab, antibiotics (ciprofloxacin and metronidazole), integrin inhibitors, and budesonide/beclomethasone.

From a clinical point of view, pain typically associated with AP was present in 358 patients (90.6%), and in one-third of cases, it was associated with nausea/vomiting (127 cases, 32.2%). Imaging was not performed in 56 patients (14%). Abdominal ultrasound was the primary imaging modality for diagnosing AP (n = 220, 55.7%), followed by CT (n = 189, 47.8%) and, less frequently, MRI (n = 63, 15.9%) and endoscopic ultrasound (EUS) (n = 12, 3.0%). When imaging was performed (n = 345), edematous pancreatitis (71.6%) or a normal pancreatic gland (23.5%) was typically described. The imaging findings are graphically presented in Supplementary Figure S3.

Testing for pancreatitis-associated genetic mutations was performed in eleven patients (2.7%), with only one positive result carrying the CFTR (cystic fibrosis transmembrane conductance regulator) gene mutation. A mild form of AP, both clinically (346, 86.9%) and radiologically (325, 95%), was usually diagnosed.

### 3.1. Drug-Induced Pancreatitis

**Figure 4:**
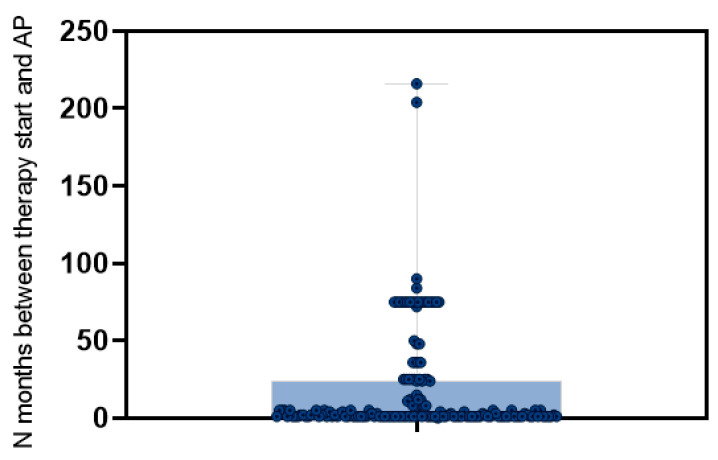
**Box and whiskers plot summarizes the interval between thiopurine therapy starting and acute pancreatitis (AP) onset.**

Drug-induced pancreatitis (DIP) usually occurred with a mild course (n = 212, 96.4%). The suspected drug was AZA/6-MP in 172 cases (77.8%). Thiopurine-induced AP occurred mainly in CD patients (n = 137, 79.6%), of whom 86 (62.7%) had active disease. Nearly 50% were smokers or ex-smokers. Overall, 43 (25%) patients received concomitant treatment with 5-ASA, 26 (15.1%) with biologics, and 8 (4.6%) with budesonide. The information about the dosage and duration of therapy with thiopurines at AZA-induced AP onset was available in 159 out of 172 (92.4%) patients: the median value was 2 mg/kg [IQR 0.5–2.5], and the median duration of therapy was one month [IQR 0.75–2] (Figure 4).

In 87.2% of patients, AZA-induced AP occurred within six months. Thus, 12.8% (n = 22) of patients undergoing AZA therapy did not fulfill the temporal criterion. Other drugs reported as possibly responsible for drug-related AP were 5-ASA (n = 35, 15.9%), antibiotics (n = 3, 1.4%), NSAIDs (n = 2, 0.9%), sulphasalazine (n = 2, 0.9%), methotrexate (n = 1, 0.4%), and steroids (n = 1, 0.4%). Information about further drug treatment was not provided. In 210 patients (95%), the suspected drug was immediately withdrawn, and most did not attempt re-administration (94.6%). In the few cases where the drug was re-administered (n = 12), nine patients (75%) experienced AP recurrence, whereas the re-administration was uneventful in three patients (1.3% of 220). An algorithm for diagnostic evaluation of suspected drug-induced pancreatitis is reported in Supplementary Table S1.

Overall, 17 (7.7%) patients had a recurrence of AP during a median follow-up period of 67 months [IQR 36–104.5]. Recurrence occurred even without re-administration of the drug in 6 of 220 patients (3.6%).

Among the 32 patients with uncertain DIP, 11 (34.3%) showed an increase in ALT. Imaging was performed in 28 cases (87.5%), with US used in 18 (64.3%), CT in 14 (50%), and MRI in 2 (7.1%). One patient eventually received a final diagnosis of AIP. In one case (0.4%), in a non-smoker and non-alcohol patient with extensive UC, progression to CP was observed during the follow-up period, despite the absence of recurrences (in this case, the suspected drug responsible for the first episode was 5-ASA).

### 3.2. Gallstones-Related Pancreatitis

In the supposed gallstones-related AP group, moderate or severe AP was reported in 18 (30.5%) and 2 (3.4%) patients, respectively. ALT levels were increased in 47 patients (79.7%), and a cholecystectomy was previously reported in 9 cases (15.2%). In 14 patients (23.7%), including 12 CD and 2 UC, surgery had been previously performed, including 10 ileocecal resections and 4 proctocolectomies with ileostomy or ileoanal pouches. Via imaging, the most frequent finding was isolated gallbladder stones (n = 36). The presence of lithiasis in the common bile duct or biliary dilation was reported in six (10.3%) and nine (15.2%) patients, respectively, regardless of the presence of gallbladder stones. The findings regarding the diagnosis of gallstone-related AP are presented in Supplementary Table S2. In 10 (16.9%) patients (see white boxes in the table), diagnostic criteria were insufficient to diagnose biliary pancreatitis.

Patients received no treatment in 8 cases (13.8%), whereas 6 (10.3%), 36 (62.1%), and 8 (13.8%) underwent ERCP, surgery, or both, respectively. Recurrence of AP was observed in four patients (6.8%) despite the endoscopic and surgical treatment (as reported in the table in brackets). In one case, evolution to CP was observed during a median follow-up of 70 months [IQR 30.75–84.5].

### 3.3. Idiopathic Pancreatitis

In 54 (13.6%) patients, the etiology of AP was unknown and reported as idiopathic. Mild AP was observed in most cases (n = 46, 85.2%), occurring before IBD diagnosis in 12 cases (22.2%), with a mean period of 2.6 years, concomitantly or within one year in 17 (31.5%), and after an established diagnosis of IBD in 25 (46.3%) cases, with a mean period of 9.7 years. When diagnosed, active IBD was reported in 17 (40.5%). In idiopathic AP, an increase in ALT was present in nine (16.7%) patients. Eight patients experienced moderate/severe AP, and three of them showed an increase in ALT without evidence of gallstones.

The diagnostic work-up for idiopathic AP included US in 28 (51.8%) and CT in 29 (53.7%) cases. MRI was performed in only 13 (24.1%) at AP onset but was used as the imaging modality of choice during the follow-up period (imaging performed at the onset and during the follow-up of idiopathic AP is reported in Supplementary Figure S4). The recurrence rate was 24.1% during a median follow-up of 54.5 months [IQR 22.25–87.5]. No evolution towards CP was observed. In one patient, AIP was eventually diagnosed.

### 3.4. Autoimmune Etiology of Acute Pancreatitis

AIP was diagnosed in thirty-three AP patients, including two patients who had been previously diagnosed with drug-induced (5-ASA) and idiopathic AP. AIP was mostly diagnosed in patients with UC (78.8%, *p* = 0.00007). AP preceded IBD diagnosis in 6 patients (18.2%), occurred within one year in 14 patients (42.4%), and followed it in 12 patients (36.4%). When already diagnosed, IBD was active in 12 (44.4%) and in remission in 15 (55.6%) cases. Cross-sectional imaging was performed in most cases. Only one patient was diagnosed after US. Supplementary Figure S5 shows the diagnostic work-up. In almost all cases, cross-sectional imaging showed pancreatic alterations typical of AIP, including pancreatic swelling (n = 29, 87.9%) and multiple main pancreatic duct strictures (n = 3, 9.1%). Less frequently observed findings include peripancreatic rims (n = 3, 9.1%), duct penetration sign or absence of upstream MDP dilation (n = 7, 21.3%), focal lesions (n = 3, 9.1%), and biliary strictures (n = 4, 12.1%). IgG4 levels were unknown/not measured in 5 (15.2%) patients, normal in 24 (72.7%), <2-fold in 3 (9.1%), and ≥2-fold upper normal value in 1 (3.0%). The ongoing therapy at AP onset was 5-ASA in 14 (42,4%), AZA in 2 (6.1%), systemic steroids in 2 (6.1%), biologics in 3 (9.1%), and others in 3 (9.1%) patients. In 13 (39.4%) cases, no treatment was being administered.

According to the ICDC, applied in 27 (81.8%) patients, definitive and probable type 2 AIP were diagnosed in 3 (9.1%) and 23 (69.7%) patients, respectively; probable type 1 AIP was diagnosed in 1 (3.0%) patient; and no definitive type 1 or type NOS AIP was diagnosed.

Steroids were successfully administered after AIP diagnosis in 23 patients (69.7%), with a dosage reported in 13 (56.5%) out of 23 patients, ranging from 0.3 to 1 mg/kg/die, for a variable duration (from 1 to 4 weeks). Recurrence was observed in thirteen patients (39.4%) after a median time of 8 months (IQR 3–33.5), including five patients without steroid treatment after a median time of 6 months [IQR 3–29], three patients treated with a low dosage or duration of steroids with a median time of 2 months, and five patients with an adequate dosage reported after a median time of 19 months [11.5–52]. In one patient (3%), evolution to CP was reported during the follow-up period.

### 3.5. Follow-Up and Outcome

Overall, during a median follow-up of 62 months [IQR 34–96], AP recurrence was reported in fifty-nine patients (14.8%), while progression to CP was reported in six (1.5%). Pancreatic cancer was diagnosed in two (0.5%) during the work-up of the AP episode, but cancer was not diagnosed during the follow-up period. Seven (1.7%) patients died during the follow-up.

The outcomes of AP in IBD patients according to different etiologies are reported in Table 3.

## 4. Discussion

The PANDORA study found a 0.58% prevalence of AP in IBD patients, lower than expected according to clinicians’ perceptions (as reported in the survey) and compared to a previously published study, where diagnostic criteria were not clearly elucidated [11,12,13,14]. To date, this represents the most extensive study concerning the association between AP and IBD, encompassing a cohort of more than 60,000 IBD patients. While the survey initially reported 780 cases, only 501 were effectively entered into the database, and 398 met the inclusion criteria. It is plausible that the perception of AP incidence in IBD patients is higher due to the general consideration of higher risk in this setting. Moreover, pancreatic enzyme elevation or atypical pain may have been mislabeled as AP and reported in subsequent medical evaluations; only the re-evaluation of single cases led to a re-definition of the condition. Despite its retrospective design, the study’s strength lies in the meticulous case-by-case analysis and the application of the Atlanta criteria for AP diagnosis. This rigorous approach ensured the exclusion of non-pancreatic abdominal pain or cases of chronic pancreatic enzyme elevation without pancreatic disease. AP primarily occurred in IBD patients under 40 years old. Differences regarding risk factors were found between patients with CD and UC. In CD patients, a higher prevalence of family history of IBD and smoking habits was observed, suggesting the possible role of interaction between genetic and environmental factors [15]. Smoking is a recognized factor worsening the clinical course of CD, probably altering cytokine expression in the intestinal mucosa [16] and triggering pancreatic damage [17]. In our study, CD patients with AP mostly had an ileal disease, with or without colonic involvement, usually with inflammatory disease behavior, consistent with the young age at the onset of IBD and disease duration. Moreover, EIMs, specifically arthropathy, and need for surgery were more frequently reported in comparison to UC patients, thus suggesting a more aggressive course of the disease with systemic impairment of immune-mediated responses. In a recent study comparing AP in IBD and non-IBD pediatric patients, arthropathy emerged as a risk factor for the development of AP [18]. Paneth cell dysfunction may play a role in this context, with changes in intestinal permeability and gut microbiota, as suggested in animal studies [19]. It is plausible that the inflammatory activity and damaged mucosa are responsible for the overexpression of cytokines (i.e., IL-33) with different targets [20]. Previous studies have focused on autoimmunity in AP occurring in IBD patients, involving the dosage of autoantibodies against pancreatic cells (PABs), reported in up to 41% of CD patients and up to 23% of UC patients in previously published studies [21]. The PABs belong to the IgG and IgA isotypes, and their target seems to be a glycoprotein predominantly expressed in the pancreas known as GP2. Recent studies showed GP2 in the epithelium of Peyer’s patches. In a Hungarian study on pancreatic autoantibodies in a large cohort of IBD patients, the prevalence of PAB was significantly more frequent in CD (41.1%) versus UC (22.7%) and was associated with complicated disease phenotype and EIMs (especially arthritis) [21]. However, in a study by Barthet et al. assessing the frequency of radiological and biological alterations in IBD patients with a previous AP episode by comparing data with IBD patients without a history of AP, no differences were found in serum levels of PABs (*p* = 0.48) [22]. Further prospective studies are needed to explore the possible association of PAB with AP occurrence, especially in CD patients. In the UC cohort, patients were slightly younger, with no risk factors, less frequent EIMs, and extensive colitis (n 102, 59%). The etiology remained unknown in about 22%, and AIP was diagnosed in 14%.

However, in both IBDs, the primary cause of AP was drug-related (55.3%), namely due to thiopurines (77.8%), especially in CD patients, whereas for other drugs, no clear evidence is available. The occurrence of AP related to AZA and its active metabolite 6-MP has been widely described as an idiosyncratic, dose-independent adverse drug reaction, usually occurring within 1–2 months from its introduction, especially in CD patients [12,22,23,24,25]. The AP occurrence is not similarly observed when thiopurines are used to treat other diseases [5,26]. The reason for this association remains unclear but is likely due to genetic predisposition and possible immune-mediated mechanisms [27,28]. Polymorphisms in the gene encoding thiopurine methyltransferase enzyme are associated with dose-dependent adverse effects, including myelosuppression and hepatotoxicity, but are unrelated to AZA-induced pancreatitis risk. Recent studies identified the association of the HLA-DQA1*02:01-HLA-DRB1*07:01 haplotype with a 17% risk of developing pancreatitis in patients homozygous for the at-risk allele. However, this association needs to be further investigated [29]. In a prospective study by Teich et al. [22], AP occurred in 7.3% of IBD patients starting AZA treatment, and smoking was the strongest risk factor for AZA-induced AP (OR = 3.2), usually mild and rapidly improved after drug discontinuation. In the present study, the median duration of therapy in drug-induced pancreatitis was one month, and in 87.2% of patients, the AZA-induced AP occurred within six months. Thus, the temporal criterion for drug-induced pancreatitis was not fulfilled in at least 12.8% (n = 22) of AZA patients. Moreover, in all cohorts with drug-induced AP, a negative rechallenge and recurrence, even without drug re-administration, were observed in 1.3% (n = 3) and 3.6% (n = 6), respectively, making the hypothesis of drug-related etiology less plausible in a total of 31 (18%) of cases. In these patients, a concomitant ALT elevation was observed in 43% of cases, raising the hypothesis of an alternative etiology of AP, namely a gallstone-related etiology. Nevertheless, the exact ALT value is unknown, and US was performed on all patients. Indeed, according to widely accepted guidelines [30], gallstone-related AP diagnoses should involve blood tests (ALT > 150 U/L) and US to reach a sensitivity of 95–98%. Gallstones are frequently observed in IBD patients, especially CD [31], with an asymptomatic presence of gallbladder stones or clinical manifestations ranging from biliary colic pain to gallstone-related AP. In the present study, gallstones were described in about 20% of CD and 15% of UC patients, respectively, and were reported as a cause of AP in 17.8% and 10.9%, respectively. About 25% were resected patients, mainly CD with ileocecal resection, probably linked to the impaired enterohepatic cycling of bilirubin [32]. Compared to other etiologies, patients were older, and AP was clinically more severe. However, when the diagnostic route is detailed, isolated gallbladder stones or absence of imaging alterations are found in about 17% of cases, making diagnosing biliary pancreatitis less plausible.

In our cohort, in 21.9% of UC and 7.1% of CD, a cause was not identified, and AP was defined as idiopathic. AP preceded IBD diagnosis in 22.2% of cases, more frequently in patients with UC compared to patients with CD. In a study recently published by Osman et al. [33] focusing on 20 idiopathic AP in IBD patients, AP is suggested to be considered an EIM that prognosticates IBD severity since a more aggressive disease course was found in patients with idiopathic AP and concomitant CD but not UC [33]. This study supports the hypothesis that idiopathic AP is compatible with an EIM of CD. In contrast, among UC patients, we have found a clinical profile of idiopathic AP patients that is compatible with the AIP group in terms of AP characteristics and recurrence rate, higher than other etiologies, in line with published data on this topic [11]. A relevant percentage of idiopathic AP may be the expression of misdiagnosed AIP (one case was eventually diagnosed in our cohort). Indeed, the absence of a serological marker for type 2 AIP (more frequently observed in IBD patients), the rarity of this condition requiring expert radiological evaluation, and the difficulty of combining ICDC due to complex histological acquisition in this setting make the diagnosis of AIP challenging. Moreover, in the absence of histological diagnosis, ICDC require an IBD diagnosis in addition to steroid response to formulate a “probable” diagnosis of autoimmune pancreatitis [7]. However, AIP may precede IBD onset with a variable interval (frequently within two years) [16,17], and diagnosis may be unpredictable. The clinical onset may occur with AP or different symptoms, including jaundice, weight loss, and abdominal pain. The diagnosis requires a combination of clinical and radiological findings through MRI. However, in the PANDORA cohort of patients, MRI is performed relatively infrequently, probably due to the mild presentation of AP and rapid recovery, but maintains an important role in the follow-up period of idiopathic AP in high suspicion of AIP and recurrence. Moreover, in about 25% of patients, ICDC are not applied, and the diagnosis of AIP is based on clinical presentation. When applied, ICDC allow one to reach a probable diagnosis. This is explained by the high rate of the diffuse form of AIP, with pancreatic swelling in most cases, and the low need to obtain histological samples necessary for diagnosing “definitive” AIP. The definitive diagnosis of type 2 AIP in patients who have not been diagnosed with IBD may raise awareness of bowel symptoms and avoid a delayed IBD diagnosis [34]. Conversely, a correct diagnosis of AIP allows one to manage the disease and start an adequate treatment. Our study observed a 39.4% recurrence rate after a median time of 8 months in AIP patients, which may be due to the low-dose steroid therapy or short-duration treatment.

The PANDORA study is the first large-scale European study on AP in IBD patients. The strength of our study was the number of participating centers, with about 500 patients evaluated against a total of about 69.000 IBD patients across Europe, the long follow-up, and the single-case revision by an expert pancreatologist. Our study has the limitation of a retrospective design, which may lead to incorrect estimates of the real number of cases, the inclusion of partial information, and the inability to revise imaging methods.

## 5. Conclusions

In conclusion, the prevalence of AP in IBD patients is lower than expected when strict diagnostic criteria are applied, facilitating the exclusion of patients with non-pancreatic pain and/or asymptomatic pancreatic enzyme elevation. Despite good adherence to diagnostic work-up, some limitations emerged, including a 14% rate of unperformed imaging, a potential 12.3% rate of missed or wrong causal diagnosis, and a rising risk of undertreatment and recurrence. The most probable cause of AP in IBD is drugs, followed by gallstones in CD and autoimmune pancreatitis in UC. However, in about 18% of drug-induced pancreatitis, the exact causality cannot be established. A correct diagnosis of AIP and an adequate steroid treatment are essential to avoid recurrences and morbidities over time.

## Figures and Tables

**Table 1:** Baseline characteristics of 398 IBD patients with AP.

	Overall	CD Patients	UC Patients	*p* Value
	N = 398	N = 225	N = 173	
Sex				
Male	191 (48%)	106 (47.1%)	85 (49.1%)	0.688
Female	207 (52%)	119 (52.9%)	88 (50.8%)	
Mean current age (in years) ± sd (range)	44.8 ± 15.3	46.8 ± 14.9	42.3 ± 15.5	0.169
	(18–91)	(19–80)	(range)	
Family history				
No	322 (80.9%)	173 (76.9%)	149 (86.1%)	
IBD	63 (15.8%)	47 (20.9 %)	16 (9.3%)	<0.00001
Pancreatic disease	6 (1.5%)	4 (1.8%)	2 (1.1%)	
Both	7 (1.8%)	1 (0.4%)	6 (3.5%)	
Smoking habit at IBD diagnosis				
Yes	99 (24.9%)	82 (36.4%)	17 (9.8%)	
No	236 (59.3%)	107 (47.6%)	129 (74.6%)	<0.00001
Ex-smokers	63 (15.8%)	36 (16%)	27 (15.6%)	
Alcohol assumption at IBD diagnosis				
No	315 (79.1%)	176 (78.2%)	139 (80.4%)	
Former drinker	6 (1.5%)	2 (0.9%)	4 (2.3%)	
Mild drinker	71 (17.9%)	45 (20%)	26 (15%)	0.605
Heavy drinker	6 (1.5%)	2 (0.9%)	4 (2.3%)	
Age at IBD onset (according to Montreal classification)				
A1 < 16 yr	24 (6%)	10 (4.5%)	14 (8%)	0.082
A2 16–40 yr	263 (66%)	144 (64%)	119 (68.9%)	
A3 > 40 yr	111 (28%)	71 (31.5%)	40 (23.1%)	
Extent of CD (according to Montreal classification)				
L1 (ileal)	-	92 (40.9%)	n/a	
L2 (colonic)	-	21 (9.3%)	n/a	-
L3 (ileocolonic)	-	111 (49.4%)	n/a	
L4 (upper GI disease) *	-	11 (4.9%)	n/a	
CD behavior (according to Montreal classification) **				
B1 (non-stricturing and non-penetrating)		126 (56%)		-
B2 (stricturing)		69 (30.7%)		
B3 (penetrating)		31 (13.8%)		
p (perianal disease)		25 (11.1)		
Extent of UC (according to Montreal classification)				
E1 (proctitis)		n/a	26 (15%)	
E2 (left-sided)		n/a	45 (26%)	-
E3 (extensive)		n/a	102 (59%)	
EIMs				
No	285 (71.6%)	152 (67.5%)	133 (76.9%)	0.04
Yes	113 (28.4%)	73 (32.5%)	40 (23.1)	
Surgery				
**Yes**	100 (25.1%)	78 (34.6%)	22 (12.7%)	
*Ileocecal resection*	*66 (66.0%)*			
*Colectomy with ileostomy*	*5 (5%)*			<0.00001
*Stricturoplasty*	*3 (3%)*			
*Colectomy with ileo-rectal anastomosis*	*3 (3%)*			
*Colectomy*				
*Proctocolectomy with ileoanal pouch*	*4 (4%)*			
*Surgery for abscesses and fistula*	*13 (13%)*			
**No**				
	*7 (7%)*			
	298 (74.9%)	147 (65.4%)	151 (87.3%)	

IBD: inflammatory bowel disease; CD: Crohn’s disease; UC: ulcerative colitis; EIMs: extraintestinal manifestations. * In 1 case, UGI disease was isolated; in 10 cases, it was concomitant with other locations. ** Overlapping behavior was present in 25 cases.

**Table 2:** Features of patients at acute pancreatitis onset in IBD patients.

	Overall	CD Patients	UC Patients	*p* Value
	N = 398	N = 225	N = 173	
Mean age in years at AP onset	37.6 ± 15.3	39.2 ± 14.9	35.5 ± 15.6	0.97
(range)	(11–85)	(11–71)	(12–85)	
Alcohol assumption at AP onset				
Unknown	59 (14.8%)	35 (15.6%)	24 (13.9%)	
None	265 (66.6%)	146 (64.9%)	119 (68.8%)	0.399
<20 g/day	57 (14.3%)	38 (16.9%)	19 (11%)	
>20 g/day	12 (3%)	4 (17.7%)	8 (4.6%)	
Binge drinking	5 (1.3%)	2 (0.9%)	3 (1.7%)	
Smoking habit at AP onset				
Unknown	14 (3.5%)	8 (3.6%)	6 (3.4%)	
No smoker	234 (58.8%)	106 (47.1%)	128 (74%)	<0.00001
Ex-smoker	44 (11%)	24 (10.7%)	20 (11.6%)	
median time from stop (in years) and [IQR]	*4.5 [1–9]*			
Active smoker	106 (26.7%)	87 (38.6%)	19 (11%)	
mean cig/day (range)	13 (5–40)			
AP onset compared to IBD				
Concomitant (within one y)	145 (36.4%)	77 (34.2%)	68 (39.3%)	
AP before IBD	37 (9.3%)	12 (5.3%)	25(14.5%)	0.001
AP after IBD	216 (54.3%)	136 (60.5%)	80 (46.2%)	
Clinical onset of AP				
Asymptomatic	17 (4.5%)	8 (3.6%)	9 (5.2%)	
Typical pain	357 (90.6%)	204 (90.7%)	153 (88.4%)	0.468
Atypical pain	19 (4.7%)	12 (5.3%)	7 (4%)	
Nausea/vomiting	127 (32.2%)	74 (32.9%)	53 (30.6%)	
Pancreatic enzyme levels				
Unknown	24 (6%)	12 (5.3%)	12 (6.9%)	
Normal	2 (0.5%)	1 (0.4%)	1 (0.6)	0.439
≤2-fold unv	30 (7.5%)	16 (71.1%)	14 (8.1%)	
>2-fold unv	342 (86%)	196 (87.2%)	146 (95.4%)	
ALT levels				
Unknown	88 (22.2%)	45 (20%)	43 (24.9%)	
Normal	213 (53.5%)	122 (54.2%)	91 (52.6%)	0.456
Elevated	97 (24.3%)	58 (25.8%)	39 (22.5%)	
Previous cholecystectomy *				
Yes	25 (6.3%)	15 (6.6%)	10 (5.8%)	0.728
No	342 (85.9%)	193 (85.8%)	149 (86.1%)	
Gallstones				
Unknown	12 (3.1%)	8 (3.6%)	4 (2.3%)	
No	313 (78.6%)	170 (75.6%)	143 (82.7%)	0.118
Yes	73 (18.3%)	47 (20.8%)	26 (15%)	
*Gallbladder stones*	*56 (76.7%)*			
*Common bile duct stones*	*9 (12.3%)*			
Indirect signs of gallstones **	*10 (13.7%)*			
Clinical severity of AP				
Mild	346 (86.9%)	194 (86.2%)	152 (87.7%)	
Moderate	41 (10.3%)	26 (11.6%)	15 (9.8%)	0.63
Severe	11 (2.8%)	5 (2.2%)	6 (3.5%)	
Etiology of AP				
Idiopathic	54 (13.6%)	16 (7.1%)	38 (21.9%)	
Drug-related	220 (55.3%)	150 (66.7%)	70 (40.5%)	
Biliary	59 (14.8)	40 (17.8%)	19 (11%)	<0.00001
Autoimmune	31 (7.8%)	7 (3.1%)	24 (13.9%)	
Alcohol-related	14 (3.5%)	4 (1.8%)	10 (5.8%)	
Dyslipidemia/metabolic	1 (0.2%)	0	1 (0.6%)	
Post-endoscopic intervention #	4 (1%)	2 (0.9%)	2 (1.2%)	
Others	15 (3.8%)	6 (2.7%)	9 (5.2%)	

AP: acute pancreatitis; IBD: inflammatory bowel disease; CD: Crohn’s disease; UC: ulcerative colitis; ALT: alanine transaminase; unv: upper normal value. * 31 missing data; ** extrahepatic or intrahepatic biliary dilation; # post-ERCP/dilation.

**Table 3:** Comparison of baseline characteristics and outcome of acute pancreatitis according to the etiology.

	DIP n = 220	Gallstone-Related n = 59	AIP N = 33	Idiopathic n = 54
CD UC	150 (68.2%) 70 (31.8%)	40 (67.8%) 19 (32.2%)	7 (21.2%) 26 (78.8%)	16 (29.6%) 38 (70.4%)
Mean Age ± SD	36 ± 13.7	47.8 ± 16.3	32.7 ± 12.9	32.9 ± 14.6
Male Female	103 (46.8%) 117 (53.2%)	29 (49.2%) 30 (50.8%)	16 (49.5%) 17 (51.5%)	23 (42.6%) 31 (57.4%)
Severity Mild Moderate/severe	213 (96.4%) 7 (3.6%)	39 (66.1%) 20 (33.9%)	32 (97%) 1 (3%)	46 (85.2%) 8 (14.8%)
Recurrence	17 (7.7%)	4 (6.8%)	13 (39.4%)	13 (24%)
Progression to CP	1 (0.4%)	1 (0.1%)	1 (0.3%)	0
Risk of misdiagnosis	14.1%	16.9%	18.2% *	18.5%

* No application of International Consensus Diagnostic Criteria. SD: standard deviation.

## Data Availability

The data underlying this article are available in the article and the online Supplementary Materials.

## Supplementary Materials

The following supporting information can be downloaded at https://www.mdpi.com/article/10.3390/medicina61091532/s1. Supplementary Figure S1: Flowchart of this study. Supplementary Figure S2: Graphical description of extraintestinal manifestations (EIMs). Supplementary Figure S3: Imaging findings on imaging performed at AP onset. Supplementary Figure S4: Pie charts representing imaging performed at idiopathic AP onset (a) and during the follow-up (b). Supplementary Figure S5: Diagnostic work-up in suspected autoimmune etiology of acute pancreatitis. Supplementary Table S1: Pancreatitis-specific drug-induced pancreatitis probability assessment scale [35]. Table S2: Combination of imaging findings for diagnosing gallstone-related AP.

## Appendix A

SURVEY FORM

1. Please provide the name and contact details of the local study coordinator at your institution
a. First name __________________________________________________________________
b. Last name __________________________________________________________________
c. Academic title/degree ________________________________________________________
d. Job title ____________________________________________________________________
2. Name of institution and department
a. Institution __________________________________________________________________
b. City ________________________________________________________________________
c. Country _____________________________________________________________________
d. Email address and phone number _____________________________________________
3. Please provide the number of IBD patients followed at your Centre, both CD and UC during a 10-
years period (January 2011–December 2020) __________________________________________
4. Please provide the overall number of IBD patients with pancreatic manifestations during a 10-year
period (January 2011–December 2020) _______________________________________________
5. Please provide the number of IBD patients with at least one episode of Acute Pancreatitis:
______________________________________________________________________________
6. Please provide the number of patients with other pancreatic disorders:
- Autoimmune pancreatitis__________________________________________________________
- Chronic pancreatitis __________________________________________________________
- Exocrine pancreatic insufficiency ___________________________________________________
- Chronic pancreatic enzyme elevation_________________________________________________
7. Is there a pancreatologist in your institute?
□ yes
□ no
8. Do you refer your patients with pancreatic manifestation to a pancreatologist?
□ yes
□ only selected cases
□ no
9. Please state who will be responsible for the data collection in this study (e.g., medical student
supervised by a gastroenterologist; PhD candidate/research fellow; dedicated resident/clinical
fellow; gastroenterologist; research nurse) _______________________________________________
10. Please state how collection of pre-, peri- and post interventional variables was performed (e.g.,
prospectively maintained database; retrospective medical record review of digital records;
retrospective medical record review of paper records; other)
