# Supplementary material for: Acute Pancreatitis in Inflammatory Bowel Disease: Results from the European Pandora Study
Source: Medicina (Kaunas). 2025 Aug 26;61(9):1532. doi: 10.3390/medicina61091532 (PMC12471949; doi:10.3390/medicina61091532)
Supplement: Supplementary file 1 [file medicina-61-01532-s001.zip › medicina-3780798-supplementary.pdf]

Supplementary Figure S1: Flowchart of this study.
Supplementary Figure S2: Graphical description of extraintestinal manifestations (EIMs).
Supplementary Figure S3: Imaging findings on imaging performed at AP onset.
Supplementary Figure S4: Pie charts representing imaging performed at idiopathic AP onset (a) and during the follow-up (b).
Supplementary Figure S5: Diagnostic work-up in suspected autoimmune etiology of acute pancreatitis.

Supplementary Figure S1

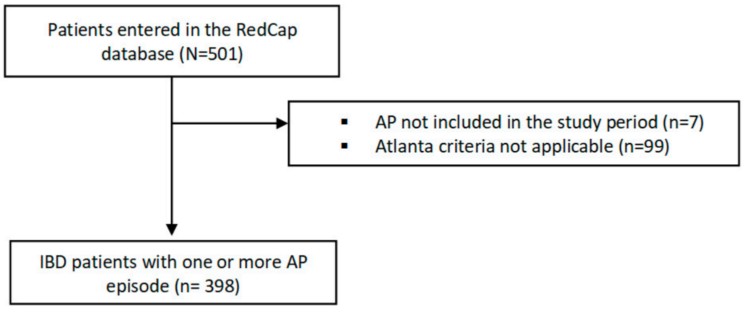

Supplementary Figure S2

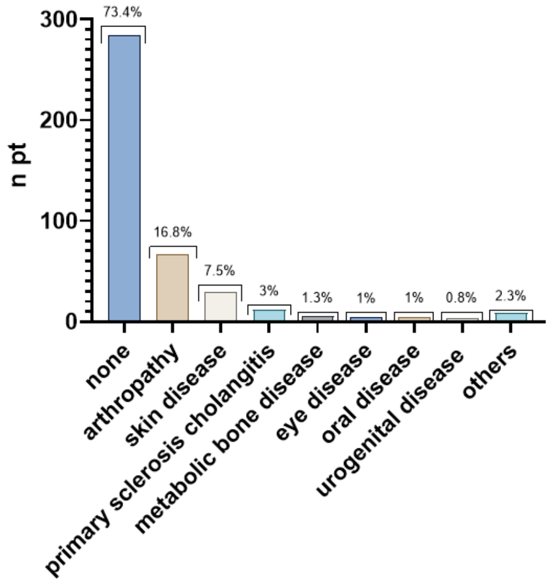

Supplementary Figure S3

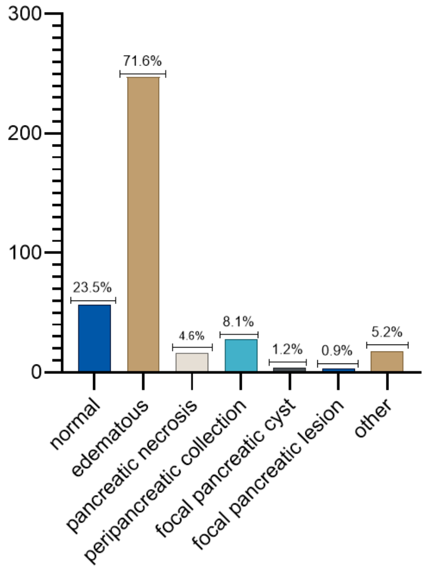

Supplementary Figure S4

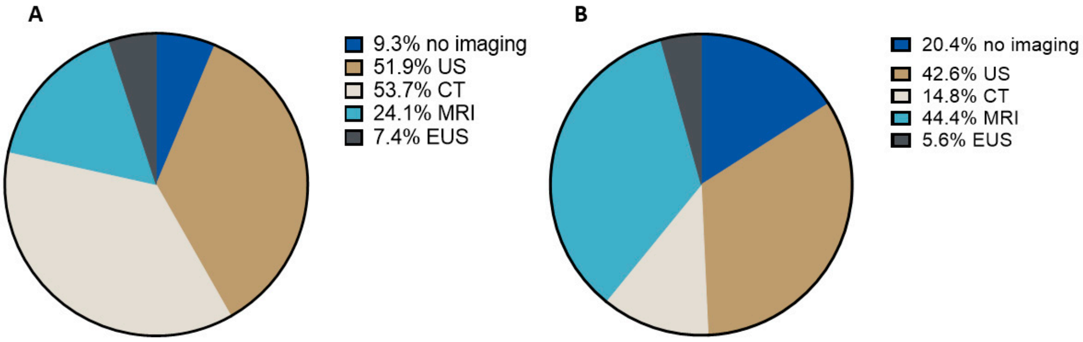

Supplementary Figure S5

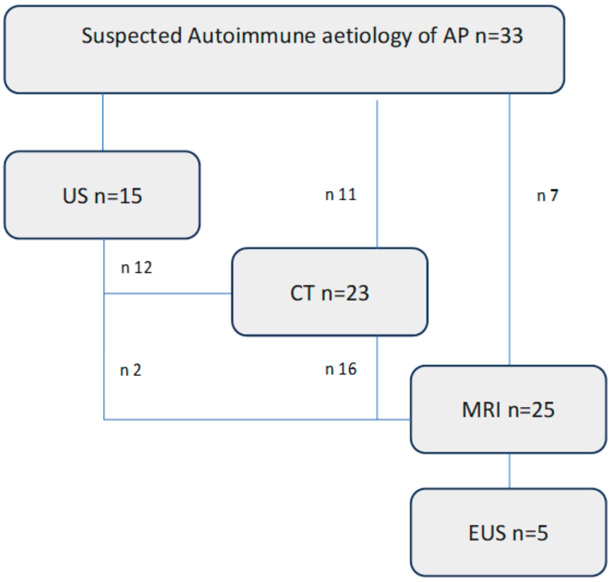

Supplementary Table S1: Pancreatitis-specific drug-induced pancreatitis probability assessment scale [35].

Question	Yes	No	Score
Are there published reports of the drug causing acute pancreatitis?	1	0	1
Was there short latency (≤7 days) between initiation of the drug and the diagnosis of acute pancreatitis?	2	0	0
Was there a temporal relationship (≤1 month) between initiation of the drug and onset of acute pancreatitis symptoms?	1	−1	0
Did the acute pancreatitis resolve following discontinuation of the drug?	1	−1	1
If a drug rechallenge was performed, did acute pancreatitis recur?	2	−1	0
Were all commonly recognised causes of acute pancreatitis ruled out? (e.g., gallstones/choledocholithiasis, alcohol, hypertriglyceridaemia, hypercalcaemia, ERCP, trauma)	1	−1	1

Question	Yes	No	Score
Was a serum IgG4 level checked? (to rule out autoimmune pancreatitis)	**1**	**0**	**1**
Does the patient have or was the patient recently diagnosed with an infection (bacterial, fungal or viral) which could cause pancreatitis?	**–1**	**1**	**1**
Was an EUS and/or MRCP performed? (e.g., to rule out occult microlithiasis, pancreatic malignancy and pancreas divisum)	**1**	**–1**	**1**
Was genetic testing (SPINK-1, CFTR and PRSS-1) performed to rule out hereditary pancreatitis? (in patients aged <30 years)	**1**	**0**	**0**
**Summative score**			**6**

Total summative score of >9: highly probable, 6–8: probable, 3–5: possible, and ≤2: doubtful.

ERCP, endoscopic retrograde cholangiopancreatography; EUS, endoscopic ultrasound; MRCP, magnetic resonance cholangiopancreatography; SPINK-1, serine protease inhibitor kazal-type 1; CFTR, cystic fibrosis transmembrane conductance regulator; PRSS-1, cationic trypsinogen gene.

Supplementary Table S2: Combination of imaging findings for diagnosing gallstone-related AP.

	Imaging findings				
**ALT levels**	*Isolated GB stones*	*CBD stones*	*Indirect signs*	*No alterations*	*Tot.*
Not performed	6	1	-	-	7
Normal	3	-	1	1	5
Elevated	27 *(r2)*	5 (r1)	8	8 (r1)	47
Tot.	36	6	9	8	59
